# Regular Sputum Check-Up for Early Diagnosis of Tuberculosis after Exposure in Healthcare Facilities

**DOI:** 10.1371/journal.pone.0157054

**Published:** 2016-06-03

**Authors:** Wen-Cheng Chao, Chieh-Liang Wu, Po-Yu Liu, Chi-Chang Shieh

**Affiliations:** 1 Department of Medical Research, Taichung Veterans General Hospital, Taichung, Taiwan; 2 Institute of Clinical Medicine, National Cheng Kung University Medical College, Tainan, Taiwan; 3 Department of Internal Medicine, Taichung Veteran General Hospital, Taichung, Taiwan; 4 Center for Quality Management, Taichung Veterans General Hospital, Taichung, Taiwan; 5 Department of Pediatrics, National Cheng Kung University Hospital, Tainan, Taiwan; Johns Hopkins Bloomberg School of Public Health, UNITED STATES

## Abstract

**Background:**

The early diagnosis of patients with TB disease is critical after an outbreak of tuberculosis (TB) infection in healthcare facilities. In this study, we report a catastrophic TB outbreak in a psychiatric healthcare facility and analyze the role of regular sputum check-ups and other diagnostic tools to facilitate an early diagnosis.

**Methods:**

Every exposed participant received regular sputum check-ups and chest X-rays (CXR) as part of the outbreak management protocol. We retrospectively analyzed data from the contact participants to identify risk factors for eventual TB development and investigated the diagnostic efficacy of regular sputum check-ups.

**Results:**

Among 133 contact participants, 16 (12.0%) developed TB during the 4-year follow-up period. Low body-mass-index (BMI) (<21) (adjusted hazard ratio (aHR) 3.16, 95% confidence interval (CI) 1.11–8.98) and long duration of contact (>3 months) (aHR 8.70, 95% CI, 1.14–63.34) independently predicted the development of TB. Even though regular sputum check-ups required significant resources, they did facilitate the early identification of new TB cases among the contact participants. Regular sputum check-ups for high-risk patients based on BMI, contact duration and CXR findings may be a practical approach when compared with universal sputum follow-up, with a slightly decreased sensitivity but high positive likelihood ratio (88%, [95% CI, 62–98%] and 5.12, [95%CI, 3.30–7.95], respectively).

**Conclusion:**

While regular sputum check-ups for all contact participants facilitated the early identification of cases after the outbreak of TB in the healthcare facility, regular sputum check-ups for high-risk patients might be an effective alternative in resource-limited settings.

## Introduction

Tuberculosis (TB) spread within a healthcare facility is a major public health problem worldwide [1]. The early identification of cases after TB exposure plays a pivotal role in preventing further transmission of *Mycobacterium tuberculosis* [2, 3]. Healthcare facilities, including facilities caring for subjects with mental illness and facilities with limited personal space, such as prisons and military camps, are characterized by an enclosed environment which leads to the inevitable close and long duration of contact between participants. These participants are hence extremely vulnerable to TB infection due to the prolonged and high-level of exposure to tuberculosis bacilli from undiagnosed patients with active TB [4–6]. The complex social, legal, and medical issues of individuals with mental illness make mandatory latent TB treatment difficult with regards to motivation for treatment, drug compliance, and potential drug interactions [7–9]. Although the early identification of new TB cases among contacts is critical to manage pulmonary TB outbreaks in psychiatric healthcare facilities [4], such a protocol is currently lacking [10]. Chest X-ray (CXR) has been reported to have a limited role in TB due to the variable radiological presentation [11] and a potential inadequate quality of CXR to precisely identify minimal pulmonary lesions [12]. In this study, we reported a severe TB outbreak in a psychiatric healthcare facility, resulting in 16 new TB cases among 133 contacts during a 4-year follow-up period. We analyzed the risk factors for the development of TB and the role of regular sputum check-ups to facilitate an early TB diagnosis among contact participants.

## Materials and Methods

### Setting

The 320-bed psychiatric healthcare facility where this study was performed is located in central Taiwan, and consists of eight sections where the patients reside according to their gender and disease activity. Of the eight sections, two acute sections (50 beds) are responsible for caring for patients with acute psychiatric stress, and their daily activities are mostly restricted to their own cubicles. In the other six chronic sections (270 beds), most daily activities are still restricted within the section, however supervised group outdoor activities are allowed for two hours per day. Given that residing in the chronic section increased the chance of contact between patients due to the group activities such as group psychotherapy and vocational rehabilitation, we divided the contacts into acute and chronic groups. Testing for human immunodeficiency virus (HIV) is routinely performed at our institute for all patients, and all of the participants in this study were HIV negative. This study was approved by the Institutional Review Board of Taichung Veterans General Hospital (SE15254A). Written informed consent from the participants for the use of their clinical records in this study was waived because this research involved no more than a minimal risk to the participants. Patient information was anonymized and de-identified prior to analysis.

### Outbreak investigation and management

In this outbreak, the index case was a 42-year-old female who was diagnosed with far-advanced pulmonary tuberculosis in July 2011 due to a strongly positive sputum smear (acid-fast stain, AFS, 4+) and bilateral large cavities (>4 cm) on CXR. Considering her significant body weight loss since October 2009, we assumed that her infectious period was from July 2009 to July 2011. During this 2-year infectious period, she stayed in the acute section for 5.5 months and the rest of the time in the chronic section.

Those exposed to the index case for cumulatively more than 40 hours in the infectious period were defined as contact participants [13]. Two contact participants were diagnosed with TB in the 2 months after the index case had been diagnosed. The Taiwanese Center for Disease Control (Taiwan CDC) thus organized a team to manage the reported TB outbreak. The infection control measures included an environmental improvement program, latent TB diagnosis and treatment for contact healthcare workers, and intensified efforts to identify cases in the contact psychiatric patients. The 9-month course of isoniazid (INH) used in Taiwan for latent TB diagnosis and treatment was not recommended by the management team for the contact patients with mental illness because of the possibility of INH-induced neuropathy or hepatotoxicity, potential drug interactions with their medications for mental illness [7, 9]. The policy of primary Bacille Calmette-Guérin vaccinations in infancy and a booster at 6–9 years of age in Taiwan also led to concerns of the effectiveness of diagnosing latent TB infection using the Mantoux tuberculin skin test alone [14, 15]. Therefore, intensified efforts to identify TB cases among the contact subjects with mental illness using regular sputum check-ups and CXR was suggested to facilitate the diagnose and treatment of secondary cases, and thereby avoid further transmission. In this study, the medical records of the patients were reviewed, and associated medical conditions, laboratory findings, serial CXR and sputum data were recorded. The diagnosis of all new TB cases was made by a TB committee based on adequate proof, which included a sputum mycobacterial culture, lung biopsy and high pleural adenosine aminohydrolase (ADA) level or the presence of characteristic TB-like lesions on CXR and chest computed tomography (CT).

### Chest X-ray examinations

During the 4-year follow-up period, CXR screening was arranged every 3 months in the first year and every 6 month in the following 4 years. The CXR were interpreted by four independent chest physicians who were blinded to the patient’s information. The CXR results were categorized as normal CXR, suspected TB lesions, any other infiltrations, or inadequate inspiration. Chest CT was indicated for the patients with suspected TB lesions without sputum evidence of mycobacteria or those who had sputum evidence but no obvious TB-like lesions on CXR.

### Regular sputum check-up

To obtain adequate sputum specimens, we used a specialized room with independent ventilation, devices to induce sputum, and ultraviolet germicidal irradiation equipment. In addition, the nurses were trained to carry out sputum induction with hypertonic saline nebulization and disinfection process under adequate personal protection, with each sputum induction procedure taking approximately 15 minutes [16]. As for CXR screening, sputum check-ups were conducted every 3 months in the first year and every 6 months in the following 3 years. All participants were taught how to obtain adequate sputum specimens using sputum induction in the first year. In the following 3 years, sputum induction was reserved for those with ongoing difficulty in producing sputum samples unaided.

### Mycobacterial culture and genotyping

The Taiwan CDC established the National Reference Laboratory of Mycobacteriology in 2004, which is responsible for formulating standard operating procedures, and assessing and regulating quality control programs of all laboratories in the mycobacterial laboratory examination network [17]. Both liquid media (ACTECTMMGITTM System (Becton and Dickinson, USA) and solid (Middlebrook 7H11) media were used in this study for sputum cultures. All of the nine cultured TB strains were sent to the Taiwan National Reference Centre for genotyping by spoligotyping and mycobacterial interspersed repetitive unit (MIRU, 10-loci) analysis [18, 19].

### Statistics

Data were presented as frequencies (n) or percentages (%) for categorical variables and as means ± standard deviations for continuous variables. Differences between the cases with and without TB were evaluated using the Student’s t test for continuous variables and chi-square tests for categorical variables. The Mann-Whitney test and Fisher’s exact test were used if the data were not normally distributed, as determined by the Kolmogorov-Smirnov test. A Cox proportional hazards regression model was used to identify variables that predicted the development of TB after controlling for age, sex, and other significant factors (P < 0.20) in univariate analysis. Statistical significance was set at a two-sided P value of less than 0.05. All data were analyzed using SPSS software version 18.0 (SPSS Inc., Chicago, IL, USA).

## Results

### Epidemiological description of the outbreak

**Fig 1:**
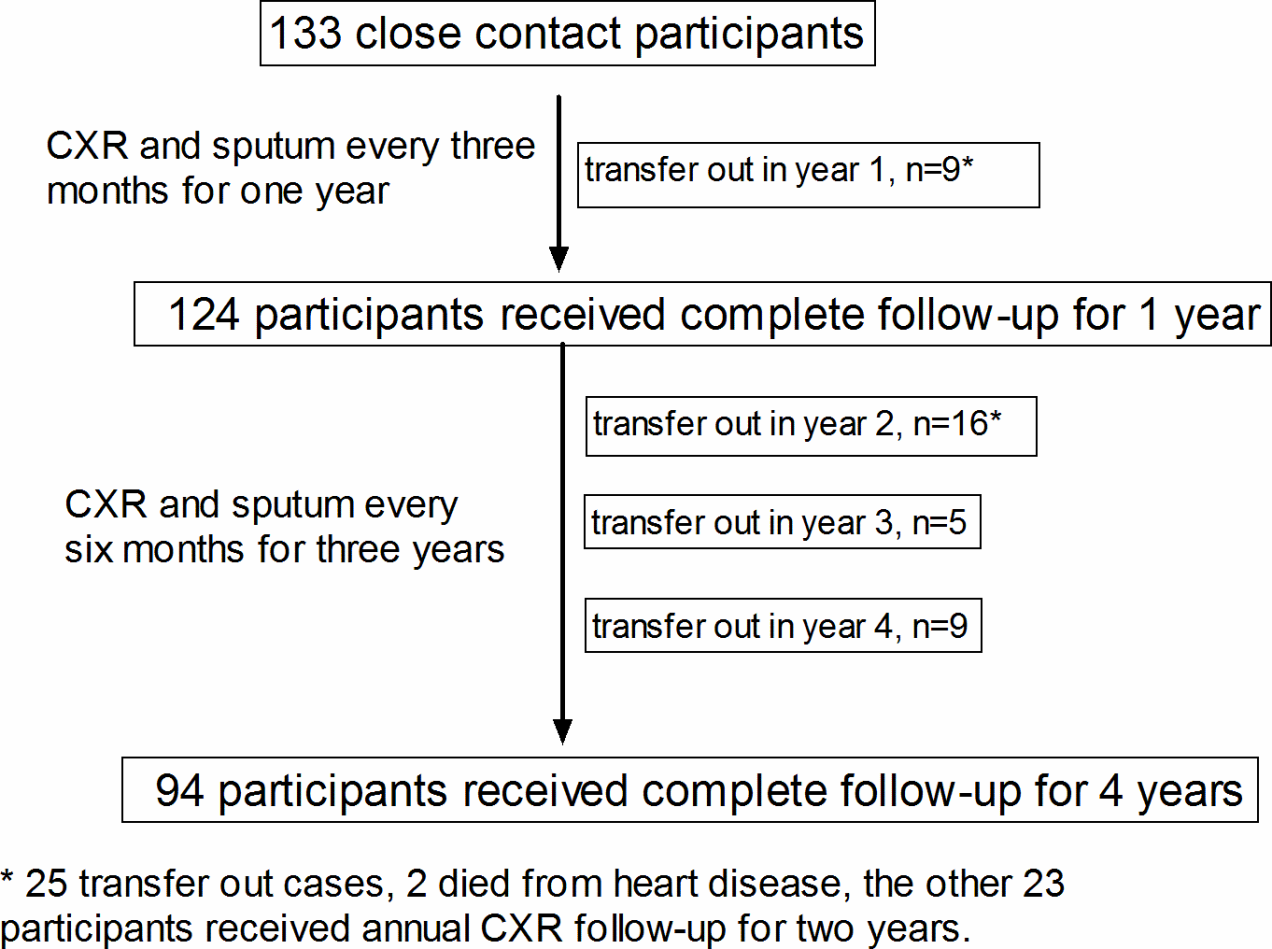
Flow chart of enrollment of participants.

A total of 133 psychiatric patients were defined as being contact participants to the index case in the outbreak investigation [20], of whom 93% (124/133) completed sputum check-ups and CXR every 3 months for 1 year. The proportions of patients completing follow-up assessments every 6 months at the end of year 2, year 3, and year 4 were 81.2% (108/133), 77.4% (103/133), and 70.7% (94/133) respectively (see S1 Dataset for details). The overall follow-up duration in this study was 3.4±1.1 years (Fig 1).

**Fig 2:**
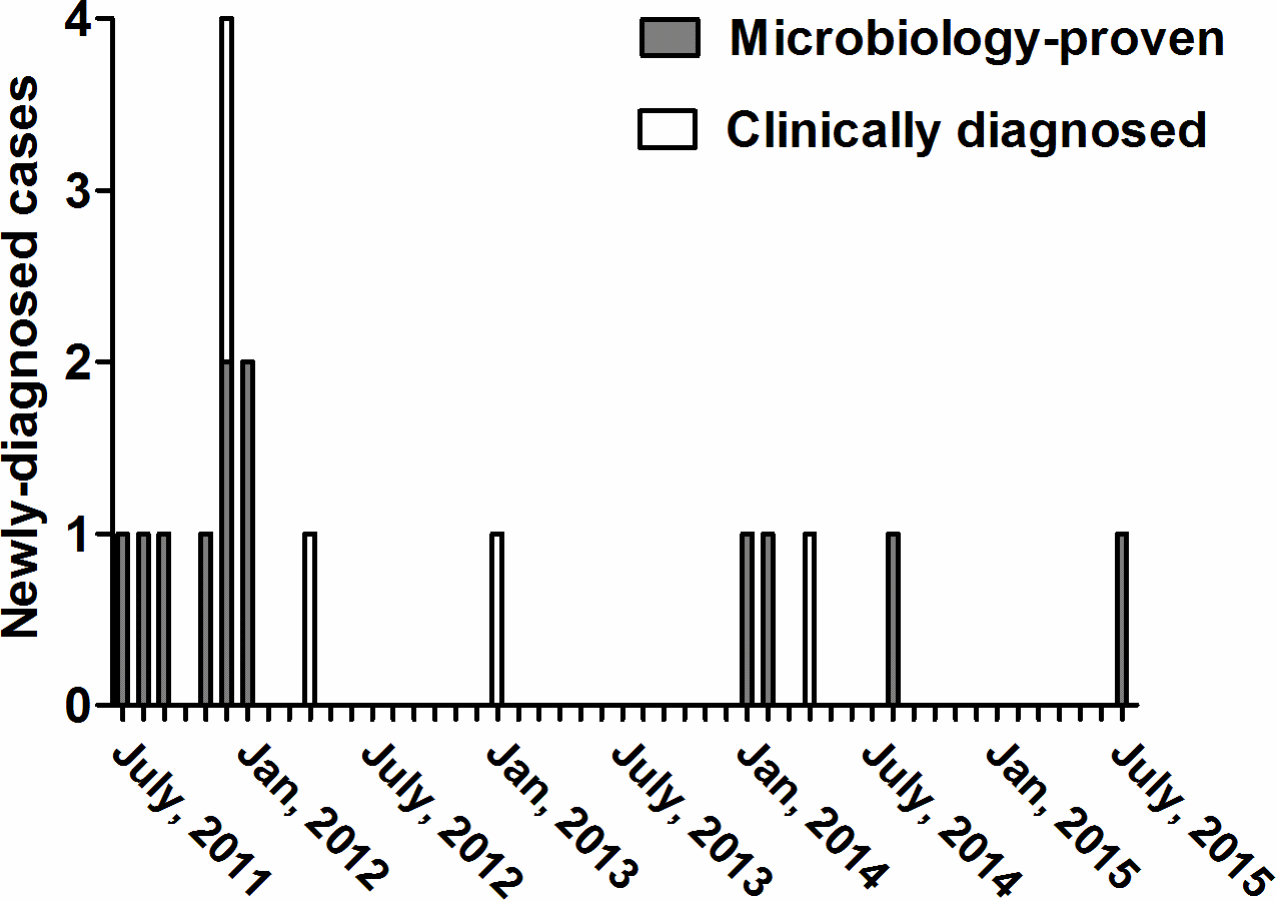
Epidemic curve of the reported TB outbreak.

Twelve percent (16/133) of the contacts developed active pulmonary TB within the 4-year follow-up period. Unlike the high grade of sputum acid-fast stain (4+) indicating a strongly contagious status of the index case, the sputum smears of the 16 new TB patients were all negative, which suggested that these 16 patients were unlikely to be other index cases. Of the 16 new TB cases, nine (56.3%) were diagnosed according to microbiological evidence including sputum mycobacteriology, one (6.3%) by caseous granuloma in a lung biopsy, one (6.3%) by a high pleural ADA level (106 IU/mL) level, and five (31.3%) by serial image findings. TB-like lesions were also found on chest CT in the patient with a high pleural ADA level, in whom pulmonary TB combined with TB pleurisy was diagnosed. Ten of the new TB patients (62.5%) were diagnosed within the first year, while the other six patients developed TB sporadically during the next 3 years (Fig 2). Genotyping analysis by the standard laboratory of the Taiwan CDC confirmed that all of the TB strains in these nine new TB patients with positive mycobacterial cultures were identical to the strain of the index case. Taken together, these results indicate that this TB outbreak was likely to have started from a single TB patient who was strongly infectious.

### Characteristics of the 16 new TB patients and the other 117 contact participants

**Fig 3:**
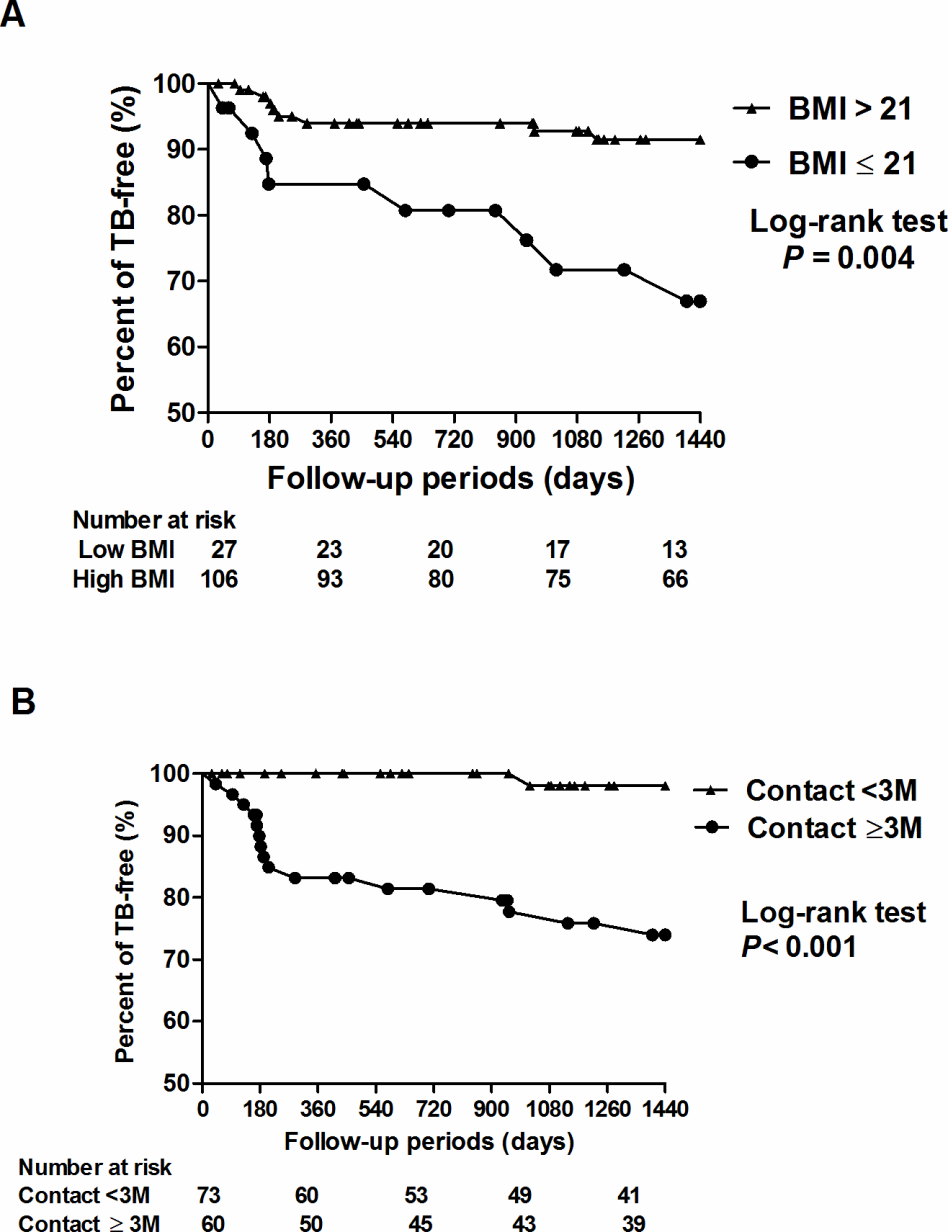
Kaplan-Meier analysis based on body mass index (A) and contact duration (B).

The clinical characteristic of all 133 contact participants are summarized in Table 1. Their mean age was 50.8±13.6 years, and 81.4% were female. The 16 patients who developed active TB had a lower body mass index (BMI) (21.9±3.4 vs.25.8±4.8, *P* < 0.001) and were more likely to be female (100% vs. 77.1%, *P =* 0.041) than the 117 participants without TB, however there were no significant differences in the other baseline demographic and laboratory data between the two groups. The duration of contact of all participants in the chronic section was 2.6±3.4 months (S1 Dataset). Compared to the 117 participants without TB, the 16 patients with TB had a longer duration of contact in the chronic section (5.4±2.6 vs. 2.5±3.5 months, *P* < 0.001), but not in the acute section (0.7±0.4 vs. 0.8±0.8 months, *P* = 0.438). Kaplan-Meier analysis demonstrated that both a low BMI (<21) and long duration of contact (>3 months) in the chronic section of the healthcare facility significantly (*P* = 0.004 and *P* < 0.001, respectively) predicted the development of TB (Fig 3). In a multivariate Cox proportional hazard regression model adjusted for age, sex, smoking and hemoglobulin, the 16 TB patients were significantly more likely to have a low BMI (<21) (adjusted hazard ratio (aHR) 3.16, 95% confidence interval (CI) 1.11–8.98) and long duration of contact in the chronic section (>3 months) (aHR 8.70, 95% CI 1.14–63.34) compared to the 117 contact participants without TB (Table 2). Taken together, these findings indicate that a low BMI and long duration of contact in the chronic section were independent risk factors for the development of TB in this study.

**Table 1:** Baseline characteristics of contact subjects^a^

Characteristics	All	TB	Non-TB	*P* value
	(N = 133)	(N = 16)	(N = 117)	
**Demographic data**				
Age (years)	50.8±13.6	50.1±9.9	50.9±14.1	*P* = 0.834
Sex (female)	107 (81.4)	16 (100)	92 (77.1)	*P* = 0.041
Body mass index (kg/m^2^)	25.4±4.8	21.9±3.4	25.8±4.8	*P* <0.001
Male	25.4±4.3	nil	25.4±4.3	nil
Female	25.4±5.0	21.9±3.4	26.0±5.0	*P* < 0.001
Diabetes mellitus	20 (15.0)	4 (25)	16 (12.0)	*P* = 0.262
Smoking	31 (23.3)	1 (6.3)	30 (25.6)	*P* = 0.117
Alcoholism	12 (9.0)	0 (0)	12 (10.3)	*P* = 0.359
Drug abuser	4 (3.0)	0 (0)	4 (3.4)	*P* = 0.595
**Laboratory data**				
WBC (cells/μl)	6692±2280	6217±1399	6757±2372	*P* = 0.377
Hemoglobulin (g/dL)	12.7±1.5	12.1±1.2	12.8±1.5	*P* = 0.058
Platelet (cells/μl)	251±81	257±65	250±83	*P* = 0.716
Fasting blood sugar (mg/dL)	103.9±44.8	110.1±57.4	103.0±43.1	*P* = 0.553
Triacylglycerol (mg/dL)	152.1±125.8	158.6±191.1	151.1±114.7	*P* = 0.826
Total cholesterol (mg/dL)	178.4±42.8	165.7±39.0	180.16±43.2	*P* = 0.209
**Contact intensities**				
Acute section (months)	0.8±0.7	0.7±0.4	0.8±0.8	*P* = 0.438
Chronic section (months)	2.6±3.4	5.4±2.6	2.5±3.5	*P <* 0.001

^a^ Data represent N (%) and mean±SD; WBC, white blood count.

**Table 2:** Cox proportional hazard regression for development of tuberculosis

Characteristics	Univariate		Multivariate	
	HR (95% C.I.)	*P* value	HR (95% C.I.)	*P* value
Age, per 1 year increment	1.00 (0.96–1.03)	0.828	0.98 (0.94–1.03)	0.471
Smoking	5.20(0.69–39.39)	0.110	4.15 (0.48–35.65)	0.195
Hemoglobulin (per g/dL decrement)	0.79 (0.60–1.04)	0.092	0.84 (0.61–1.15)	0.288
BMI < 21				
No	1.00		1.00	
Yes	4.24 (1.59–11.3)	0.004	3.16 (1.11–8.98)	0.031
Long duration of contact in chronic section (>3 months)				
No	1.00		1.00	
Yes	17.9 (2.36–135.38)	0.005	8.70 (1.14–63.34)	0.037

HR: Hazard ratio; C.I.: Confidence interval; BMI: Body mass index.

### Diagnostic efficacy of regular sputum check-ups for the early detection of new TB cases

**Fig 4:**
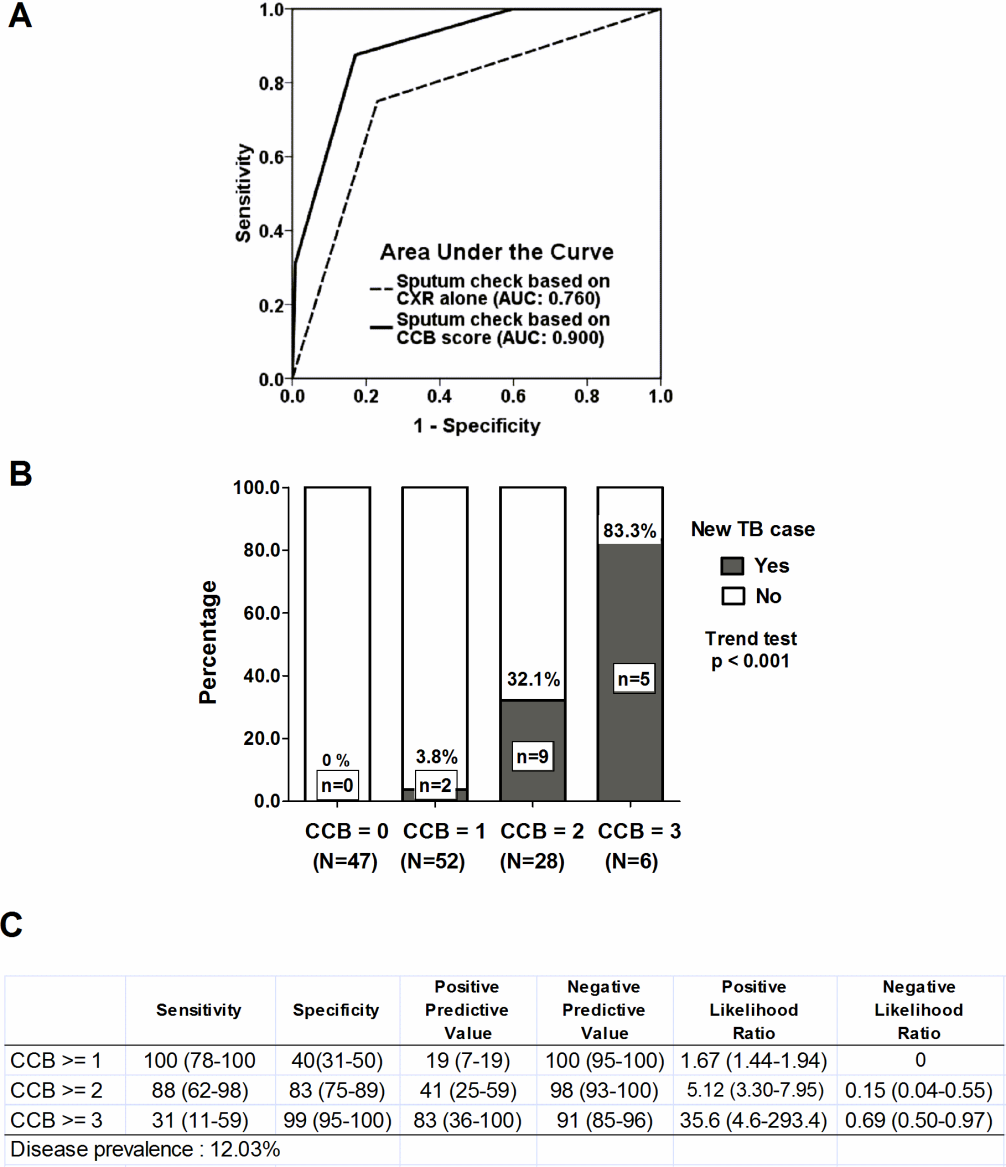
Using sputum check to early detect new tuberculosis cases based on CXR alone or combinational CCB score. Receiver operator characteristic curve (A). Separate percentage (B) and diagnostic accuracy (C) by CCB score. CCB score: CXR, Contact-duration and BMI score.

A sputum mycobacterial culture is the diagnosis basis of pulmonary TB infection, and sputum check-ups with adequate sputum samples are critical to facilitate the early identification of patients with minimal pulmonary TB lesions [21]. Interestingly, of the nine patients with positive sputum cultures for *M*.*tb*, only three (33.3%) were interpreted as having "suspected TB lesions", with two (22.2%) as "increased infiltrations" and four (44.4%) as "normal CXR" (Table 3). The six TB patients without "suspected TB lesions" on CXR underwent chest CT, and only minimal infiltrations (less than 1 cm in diameter) were found. These findings indicate the importance of regular sputum check-ups in TB outbreaks. However, unlike regular CXR, a basic and convenient tool for the diagnosis of TB, regular sputum check-ups including smears and cultures are much more resource-intensive, and we thus sought to develop a sputum check-up strategy which could be used in a setting with limited resources [22, 23]. We first analyzed the efficacy of regular sputum check-ups only for high-risk patients based on BMI, contact duration, and initial CXR findings. We integrated CXR (normal: 0; increased infiltrations or suspected TB lesion: 1), long duration of contact (<3 months: 0; > = 3 months: 1), and low BMI (> 21: 0; < = 21: 1) into an index which we termed the CCB score. Using receiver operating characteristic (ROC) analysis, the discriminatory power was better in the reserved sputum check-ups based on CCB score (the area under curve (AUC) was 0.900 [95% CI, 0.830–0.970]) for predicting the development of TB among the contact participants compared with the sputum check-ups based on CXR findings alone (AUC: 0.760 [95% CI, 0.629–0.890]) (Fig 4A). We also analyzed the separate percentages (B) and diagnostic accuracy (C) categorized by the CCB score (Fig 4B and 4C). In a resource-limited setting, a CCB score > = 2, which represented reserving sputum check-ups for those who had normal CXR findings but two of the other risk factors or those who had any infiltrations on CXR with one of the other two risk factors, had a slightly decreased sensitivity (88%, [95% CI, 62–98%]) but high positive likelihood ratio (5.12, [95%CI, 3.30–7.95]) than those with a CCB score > = 1, and a CCB score > = 2 may be acceptable to allow for the early detection of TB patients among contact participants. Among the 99 participants with a CCB score < = 1 in this study, regular sputum check-ups only detected two (2%) TB cases early, whereas of 34 participants with a CCB score > = 2, 14 (41%) TB cases were diagnosed as having TB disease, and 20 (59%) had no evidence of TB disease in regular sputum follow-up (Fig 4B).

**Table 3:** Characteristics of the 16 newly diagnosed TB cases

No	Age	Sex	BMI	Contact	Diagnosis	Diagnosis basis	CXR finding
				(months)	date		
1	60	F	18.2	5.5	8-Aug-11	Lung biopsy (caseous granuloma)	Suspected TB lesion
2	52	F	21.8	5.5	29-Sep-11	Sputum mycobacteriology^a^	Increased infiltration
3	40	F	20.3	5.5	2-Nov-11	Sputum mycobacteriology^a^	Suspected TB lesion
4	50	F	22.2	5.5	5-Dec-11	Pleural effusion (ADA: 106 IU/mL)	Suspected TB lesion
5	35	F	19.7	10.5	14-Dec-11	Sputum mycobacteriology^a^	Normal CXR^b^
6	48	F	16.8	5.5	22-Dec-11	Serial CXR and CT findings	Suspected TB lesion
7	61	F	22.5	5.5	26-Dec-11	Serial CXR and CT findings	Suspected TB lesion
8	48	F	31.1	10.5	4-Jan-12	Sputum mycobacteriology^a^	Normal CXR^b^
9	54	F	25.0	10.5	20-Jan-12	Sputum mycobacteriology^a^	Increased infiltration
10	35	F	22.8	7.5	11-Apr-12	Serial CXR and CT findings	Suspected TB lesion
11	50	F	20.7	3.5	24-Jan-13	Serial CXR and CT findings	Suspected TB lesion
12	62	F	19.3	5.5	14-Jan-14	Sputum mycobacteriology^a^	Normal CXR ^b^
13	42	F	25.8	7.5	6-Feb-14	Sputum mycobacteriology^a^	Normal CXR ^b^
14	48	F	20.0	0.5	11-Apr-14	Serial CXR and CT findings	Suspected TB lesion
15	46	F	24.0	5.5	7-Aug-14	Sputum mycobacteriology^a^	Suspected TB lesion
16	71	F	20.7	3.5	14-Jul-15	Sputum mycobacteriology^a^	Suspected TB lesion

^a^ identical strain compared with index case

^b^ minimal lesion less than 1cm was found by chest CT

F: female; ADA: adenosine aminohydrolase.

## Discussion

A TB outbreak in a healthcare facility is an important public health issue, and particularly challenging in a psychiatric healthcare facility. In this study, we described a catastrophic TB outbreak with 16 new cases during a 4-year follow-up period. We found that a low BMI and long duration of contact were key risk factors for TB infection. We also analyzed the efficacy of regular sputum check-ups to facilitate an early diagnosis.

The incidence of TB among the contact psychiatric patients was high in this TB outbreak. Sixteen new TB cases among 133 participants were identified in the 4-year follow-up period (3007.5 per 100, 000 person-year), which is 67.2 times higher than the age-matched average incidence of TB in Taiwan (44.5 new TB cases per 100, 000 person-years). One meta-analysis reported that in countries with an intermediate incidence of TB (50–100/100,000 population), the risk of TB among healthcare workers was higher than that among the general population by approximately 2.45 times [24], and that the type of contact including group activities had a great impact on TB transmission [25, 26]. The intensity of contact should be much stronger between patients in a psychiatric healthcare facility when compared with that between healthcare workers. Moreover, different contact behavior among participants may also lead to difference in TB transmission. As shown in this study, the development of TB was highly associated with the duration of contact in the chronic section, but this association was not found with contact in the acute section (Table 1 and Fig 3B). In addition, the prolonged contact with the index case with active TB in the chronic section for 2.6 ± 3.4 months (Table 1) appears to be a critical factor for the extremely high TB incidence. In this outbreak, clinical factors including the poor subjective expression ability of the index case, mistaking body weight loss as psychological stress by staff members, and systemically missing routine CXR screening due to inter-sectional transfer led to the delayed identification of the index case. This highlights the difficulty in implementing infection control measures in a psychiatric care facility [1].

Interestingly, both a low and high BMI have been reported to be associates with the development of TB [27, 28]. A 5-year cohort study investigating 1,695 adult patients with pulmonary TB in rural India reported that 80% of the female and 67% of the male patients had moderate to severe malnutrition (BMI <17 kg/m^2^) [29]. Severe malnutrition (BMI <17 kg/m^2^) was uncommon in our patients as evidence by the high BMI (25.4±4.8 kg/m^2^); however, moderate malnutrition (BMI <21 kg/m^2^) was still found to be an independent risk factor for the development of TB among the contacts in this study (Fig 3A). Although the exact cut-off value of BMI in other settings is unclear, a low BMI was still a risk factor for the development of TB disease in our patients. Consistent with these findings, a higher BMI in metabolically normal participants has also been reported to be a protective factor for TB infection [28]. However, such a protective effect has not been found in participants with a high BMI with abnormal metabolism and patients with diabetes mellitus [30]. The complex association between BMI and TB highlights the potential interaction between metabolic signaling and immune response against *Mycobacterium tuberculosis*. In our previous study, we found decreased toll-like receptor-induced interferon-γ, interleukin-6 and tumor necrosis factor-α secretions and elevated interleukin-10 secretion by leukocytes in severely obese participants (BMI 37.4 ±1.6 kg/m^2^) [31]. We also further demonstrated that resistin suppresses mycobacterium-induced inflammasome activation through the inhibition of reactive oxygen species production [32]. These results highlight the importance of nutritional state in the defense against mycobacterial infection. Both overnutrition-induced metabolic syndrome and undernutrition common in psychiatric patients may lead to susceptibility to TB infection. Restoring a normal metabolic state by appropriate nutritional management may therefore be an important issue in the control of TB [33].

CXR is a convenient diagnostic tool for TB, however it has been previously reported to be limited in sensitivity for the early identification of TB patients with mild lung lesions [11, 34]. This finding is consistent with the four patients in the current study with positive sputum cultures for *M*.*tb* but who had "normal CXR" findings (Table 3). Chest CT, a more sensitive imaging tool than CXR, has been used as a screening tool for the early identification of active TB cases in TB contact investigations among young soldiers [6]. However, some concerns including cost, radiation exposure, and case-definition in managing patients with non-specific fibrotic or infiltrative pulmonary lesions remain. Therefore, we used chest CT as an adjunctive imaging tool and used regular CXR as the screening tool in this study.

Adequate sputum sampling is the basis of diagnosing TB [21], and sufficient training to obtain adequate sputum samples is particularly crucial for patients with mental illness [35]. Sputum induction has been proven be a well-tolerated, low-cost technique with a similar diagnostic yield to bronchoscopy in the diagnosis of smear-negative pulmonary TB [36]. Adequate sputum sampling to allow for the early diagnosis of new TB cases is considered to be critical to prevent TB transmission in the beginning of an outbreak since latent TB treatment is infeasible. Indeed, to treat contact subjects with latent TB infection is an effective strategy to stop further transmission of TB in the management of TB outbreak in healthcare facilities [1], while to stop the transmission of TB is particularly critical if latent TB screening and treatment is infeasible. The results of this study provide clinical evidence of using regular sputum check-ups via induction for all contact subjects to early diagnose subjects of TB with minimal lung lesions, and this strategy may be considered to be one of the strategies in stopping further transmission of TB. Our data show that regular sputum check-ups for all high-risk contact participants for up to 4 years were an effective and necessary strategy for the early identification of new TB cases. The use of sputum induction and thorough training for how to obtain sputum samples may at least partly explain the detection of TB patients with minimal lung lesions in this study. However, unlike regular CXR follow-up, continuous regular sputum smear and culture check-ups as in the follow-up protocol used in this study is quite resource-intensive. We thus used our data to analyze the efficacy of focusing resources on high-risk subjects after a two-year regular sputum check-ups, which might be a potential alternative approach in resource-limited settings. Our data showed that 68.8% (11/16) of the TB cases were diagnosed within the first 2 (1–2) years, with the other 31.2% (5/16) of TB cases diagnosed in the next 2 (3–4) years. It is worth noting that all of the cases diagnosed in the 3–4 year period were high-risk patients, of whom 80% (4/5) had a long duration of contact (5.5 ± 1.6 months) and 20% (1/5) had a low BMI (20.0 kg/m^2^) (Table 3). Therefore, regular sputum check-ups for all contact participants for the first 2 years while reserving regular sputum check-ups only for high-risk participants may be a reasonable and resource-saving strategy in clinical practice.

There are several limitations to this study. First, the number of cases is relatively small, however the studied population was generally stable, which enabled us to provide clinical evidence of the gradual development of TB disease among the contact participants during the 4-year period and to investigate the diagnostic efficacy of measures to early identify patients with TB disease. Second, the current findings of a long duration of contact and low BMI as risk factors for the development of TB may be specific to the particular set of circumstances in this study, and further validating studies are needed to determine the cut-off value of CCB scores in other settings. Third, the quality of sputum was not measured, although the quality should have been acceptable after sputum induction and thorough training for how to obtain adequate sputum samples.

In conclusion, the findings of this study suggest that regular sputum check-ups may be used a management tool for TB outbreaks in a healthcare facility. We found that a low BMI and long duration of contact predicted the development of TB. In addition, regular sputum check-ups for all contact participants contributed to the early identification of cases, and regular sputum check-ups for high-risk participants might be an alternative practical approach in resource-limited settings.

## Supporting Information

S1 Dataset (XLS)

## Abbreviations

ADA: adenosine aminohydrolase
AFS: acid-fast stain
AUC: area under curve
BMI: body-mass-index
CCB score: integrated CXR, contact-duration, and BMI score.
CI: confidence interval
CT: computed tomography
CXR: chest X-ray
HIV: Human immunodeficiency virus
INH: isoniazid
MIRU: mycobacterial interspersed repetitive unit
Taiwan CDC: Taiwanese Center for Disease Control
ROC: receiver operating characteristic
TB: tuberculosis
